# A new species and two newly-recorded species of the genus *Micrencaustes* (Coleoptera, Erotylidae) from China

**DOI:** 10.3897/BDJ.12.e134304

**Published:** 2024-11-07

**Authors:** Yuhang Yang, Xiaoxiao Zhang, Jing Liu, Jing Li

**Affiliations:** 1 College of Plant Protection, Hebei Agricultural University, Baoding, China College of Plant Protection, Hebei Agricultural University Baoding China

**Keywords:** Encaustini, beetle, description, key, taxonomy

## Abstract

**Background:**

The genus *Micrencaustes* Crotch, 1876 (Coleoptera, Cucujoidea, Erotylidae, Erotylinae, Encaustini) includes 44 known species worldwide, 11 species having been reported to occur in China. In recent years, species of genus *Micrencaustes* in China are constantly being discovered, mainly distributed in southern China.

**New information:**

A new species, Micrencaustes (Mimencaustes) occulta **sp. nov.**, is described and illustrated. Two species, Micrencaustes (Mimencaustes) divisa Arrow, 1925 and Micrencaustes (Micrencaustes) navicularis Arrow, 1921 are recorded from China for the first time. The morphological characteristics of adults of new species are re-described in detail and illustrated. A key to Chinese species of the genus *Micrencaustes* is provided. The specimens of new species was collected from Yunnan Province and the specimens of Micrencaustes (Mimencaustes) divisa Arrow, 1925 and Micrencaustes (Micrencaustes) navicularis Arrow, 1921 were collected from Hainan Province and Guangdong Province and deposited in the Museum of Hebei University and Institute of Zoology, Chinese Academy of Sciences.

## Introduction

The genus *Micrencaustes* Crotch, 1876 (Coleoptera, Cucujoidea, Erotylidae, Erotylinae, Encaustini) was established by Crotch (1876) with *Encausteslunulata* (MacLeay, 1825) as the type species. In this genus, 44 species have been reported (Chûjô and Chûjô 1989, Chûjô and Chûjô 1990, Li 2006, Li and Ren 2006, Wegrzynowicz 2007, Li et al. 2017) which are mainly distributed in Palearctic, Oriental, Afrotropical and Australasian realms. So far, 11 species have been reported to occur in China: Taiwan, Hainan, Guangxi and Yunnan (Araki 1941, Chûjô 1968, Chûjô and Chûjô 1989, Chûjô and Chûjô 1990, Osawa and Chûjô 1990, Li 2006, Li and Ren 2006, Wegrzynowicz 2007, Meng et al. 2014). *Micrencaustes* can be distinguished from other genera by the following characters: body size medium to large, non-parallel at both sides; base of elytra and pronotum subequal in width; lacinia with two teeth at apex, maxillary terminal palpomere extremely transverse; submentum with ridges at both sides; with expressed marginal border on each side, prosternal lines present, postmesocoxal lines and postmetacoxal lines present or absent (Crotch 1876, Li 2006). This genus includes two subgenera, the subgenus Mimencausteshaving mesocoxal lines, whereassubgenusMicrencaustes does not. In this article, Micrencaustes (Mimencaustes) occulta **sp. nov.** and Micrencaustes (Mimencaustes) divisa Arrow, 1925 belong to the subgenusMimencaustes and Micrencaustes (Micrencaustes) navicularis Arrow, 1921 belongs to the subgenusMicrencaustes.

During our examination of the specimens of *Micrencaustes* from southern China, a new species and two newly-recorded species for China were discovered. A key to the species of *Micrencaustes* from China is provided.

## Materials and methods

Specimens were softened in warm water for 12 hours. Then, the whole abdomen and genitalia were detached from the body. Male and female genitalia were placed in 5% sodium hydroxide (NaOH) solution for 5 minutes, then cleaned with distilled water. Morphological characters were observed using a Motic SMZ-168 stereomicroscope and a detailed description was provide. Photographs were taken with Olympus E-M5 II camera and processed with Adobe Photoshop 2021. Morphological terminology follows Lawrence (Lawrence et al. 2010, Lawrence et al. 2011).

All specimens of this study were deposited in the Museum of Hebei University (MHBU) and Institute of Zoology, Chinese Academy of Sciences (IZAS).

## Taxon treatments

### Micrencaustes (Mimencaustes) occulta

Yang & Li sp. nov.

0EDA08AB-1278-5ABA-8D07-02E25C9A01AA

DC5BD9C5-D61F-48E2-A25C-5B3A39BD98C9

#### Materials

- **Type status:** Holotype. **Occurrence:** individualCount: 1; sex: 1 male; occurrenceID: E26B1B82-1138-5DBE-BCC0-4B69A840C898; **Location:** country: China; stateProvince: Yunnan; county: Yingjiang; verbatimCoordinates: 24.691355°N, 97.943483°E; **Identification:** identifiedBy: Yibing Ba; **Event:** year: 2012; month: 8

#### Diagnosis

Body elongate-oval, convex dorsally, black, moderately lustrous. Each elytron with two orange marks and with seven distinct striae. Clypeofrontal sulcus incomplete. Interocular distance about 0.47 times width of head. Antennae long, almost extending to the posterior edge of pronotum, antennomere 3 long and apex slightly swollen, 1.6 times as long as antennomere 4, antennomere 9 almost equal in length and width; antennomere 10 transverse, 2.1 times as wide as long. Maxillary terminal palpomere transverse, nearly semicircular, 2.8 times as wide as long. Lacinia with two teeth at apex. Pronotum with three indistinct teardrop-shaped orange marks and with a few coarse punctures on each side of base. Postmesocoxal lines absent.

#### Description

**Figure 1.**
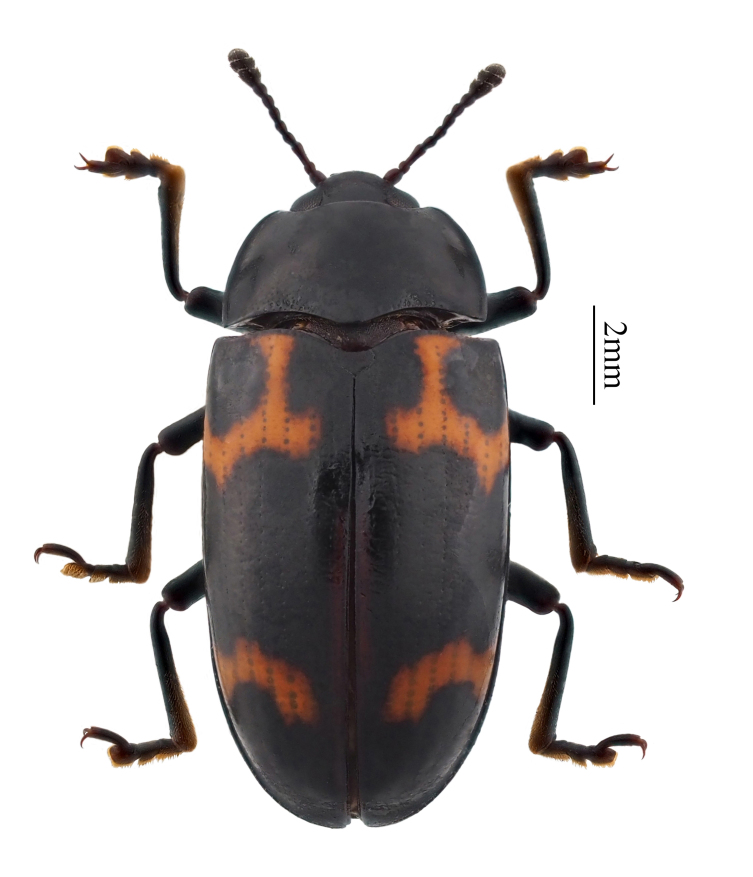
Micrencaustes (Mimencaustes) occulta **sp. nov.**, Yunnan, China.

**Figure 2.**
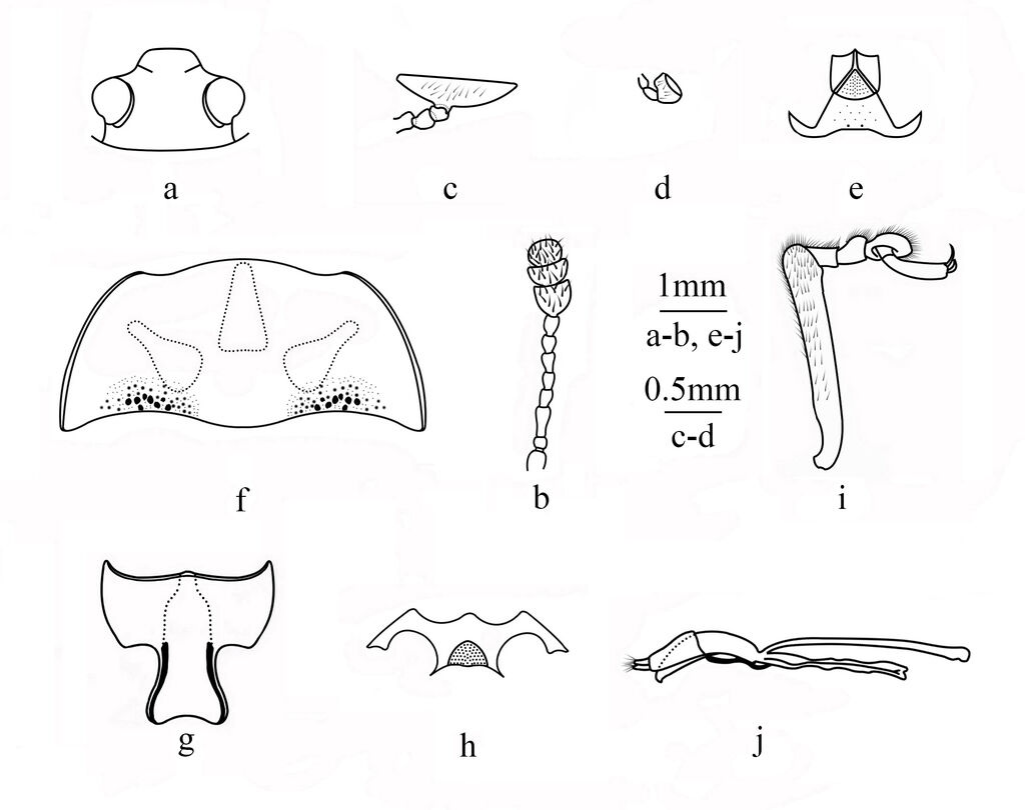
Morphological characters of Micrencaustes (Mimencaustes) occulta **sp. nov.** a head; b antenna; c maxillary palpus; d labial palpus; e mentum and submentum; f pronotum; g prosternum; h mesoventrite; i protibia and protarsus; j aedeagus. Scale bars: 1.0 mm (a-b, e-j), 0.5 mm (c-d).

Body length: 13.2 mm; width: 6.2 mm, elongate-oval, convex in lateral view, general colour black, moderately lustrous. Pronotum with three indistinct teardrop-shaped orange marks. Each elytron with two orange marks, the first mark reaching the basal edge and with a wave at posterior edge; the second mark at basal three fourths, transverse and curved, with a wave at anterior edge (Fig. 1). Head (Fig. 2a) coarsely punctured on vertex. Clypeus finely punctured, anterior edge straight. Clypeofrontal sulcus incomplete. Eyes large, coarsely facetted; interocular distance about 0.47 times width of head. Antennae (Fig. 2b) long, almost extending to posterior edge of pronotum, covered with golden setae; antennomere 1 swollen, barrel-shaped; antennomere 2 spherical; antennomere 3 long, slightly swollen at apex, 1.6 times as long as antennomere 4; antennomere 9-11 transverse and compact, forming the antennal club, antennomere 9 expanded apically; antennomere 10 crescent-shaped, 2.0 times as wide as long; antennomere 11 nearly fan-shaped; relative lengths of antennomeres 2-11: 1.5: 3.6: 2.1: 2.2: 2.3: 2.2: 2.2: 2.8: 2.0: 2.2. Maxillary terminal palpomere (Fig. 2c) transverse, strongly expanded, nearly triangular, 2.8 times as wide as long. Labial terminal palpomere (Fig. 2d) dolabriform. Mentum (Fig. 2e) small, nearly pentagonal, with middle area triangularly depressed; submentum (Fig. 2e) nearly trapezoidal, with a few coarse punctures in the middle and two extremely coarse punctures at the base.

Pronotum (Fig. 2f) 2.2 times as wide as long, nearly trapezoidal, convex dorsally, with extremely sparse punctures. Anterior edge curved opposite the head; lateral edge curved, with expressed border and slightly narrowing apically; basal edge weakly sinuate, with a few coarse punctures on each side of base. Anterior angles blunt and slightly protruded, posterior angles almost rectangular. Scutellar shield sparsely punctured, nearly heart-shaped, 1.6 times as wide as long. Each elytron with seven distinct striae, intervals sparsely punctured.

Prosternum (Fig. 2g) finely and sparsely punctured, covered with fine and short setae, anterior edge narrow, posterior edge emarginated. Prosternal process nearly long bell-shaped, prosternal process distinctly and abruptly expanded apically, apical edge of prosternal process emarginated, only slightly extending beyond anterior edge of mesoventrite. Mesoventrite (Fig. 2h) sparsely punctured, with a triangular depression in middle of posterior edge . Metaventrite finely and sparsely punctured and two elongated transverse depressions at posterior edge. Abdomen sparsely punctured, covered with short setae.

Legs (Fig. 2i) with tibiae gradually widening to apices, tibiae and tarsus covered with golden setae.

Male genitalia (Fig. 2j) with median lobe slightly curved, median strut straight, apex slightly wide, 2.3 times as long as median lobe.

#### Etymology

The species is named for the three indistinct teardrop-shaped orange marks on the pronotum, the marks seen when strong light shines on the pronotum.

#### Distribution

Micrencaustes (Mimencaustes) occulta Yang & Li **sp. nov.** is recorded in Yingjiang County, Yunnan Province, China.

#### Notes

Micrencaustes (Mimencaustes) occulta **sp. nov.** is similar to Micrencaustes (Micrencaustes) lunulata (Macleay, 1825) due to the body and colour. We examined type specimens of Micrencaustes (Micrencaustes) lunulata (Macleay, 1825) and laboratory collected specimens, these two species being distinguished by the combination of the following characters: Micrencaustes (Mimencaustes) occulta **sp. nov.** without postmesocoxal lines, each elytron with seven distinct striae, pronotum with a few coarse punctures on each side of the base, body moderately lustrous; Micrencaustes (Micrencaustes) lunulata (Macleay, 1825) with postmesocoxal lines, each elytron with eight distinct striae, pronotum with evenly fine punctures, body shiny.

### Micrencaustes (Mimencaustes) divisa

Arrow, 1925

F7EEE37A-1936-521C-A9B8-F18655AE9C74

Micrencaustes (Mimencaustes) divisa Arrow, 1925 - Arrow 1925: 79

#### Materials

- **Type status:** Other material. **Occurrence:** recordedBy: Yibin Ba and Juntong Lang; individualCount: 1; sex: 1 female; occurrenceID: 73A9E8EC-433E-50B9-9FD7-13AA98C4F963; **Location:** country: China; stateProvince: Hainan; county: Baisha; locality: Yuanmen Town; verbatimCoordinates: 19.158191°N, 109.486479°E; **Event:** year: 2007; month: 5

#### Distribution

**Figure 3.**
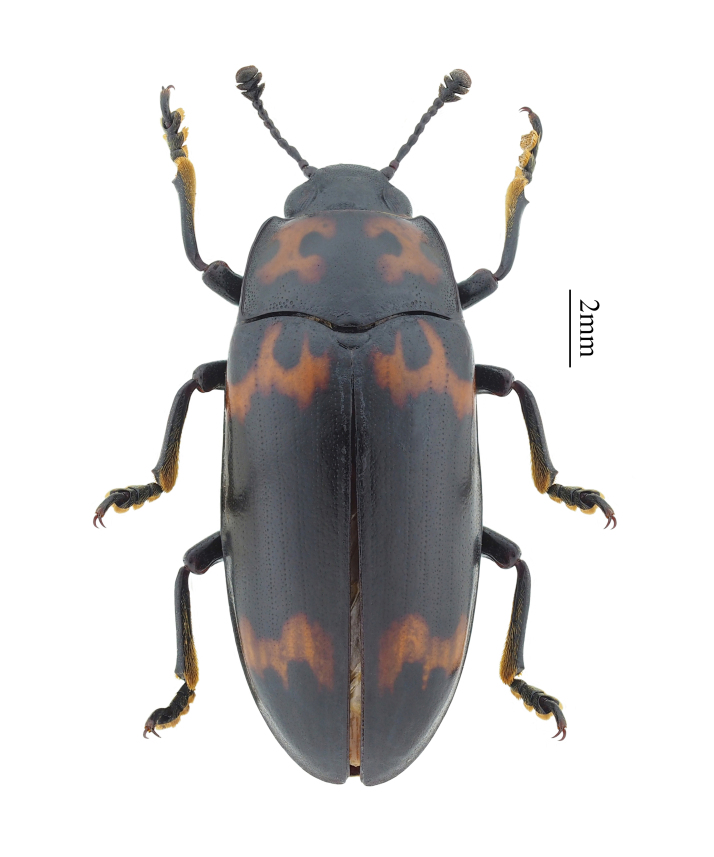
Micrencaustes (Mimencaustes) divisa Arrow, 1925. Specimen from Hainan, China.

Micrencaustes (Mimencaustes) divisa Arrow, 1925 (Fig. 3) is recorded in Yuanmen Town, Baisha County, Hainan Province, China and is also distributed in Myanmar (Arrow 1925, Chûjô and Chûjô 1989).

### Micrencaustes (Micrencaustes) navicularis

Arrow, 1922

1233087D-653B-5EC3-80FD-B5471A8F4A61

Micrencaustes (Micrencaustes) navicularis Arrow,1922 - Arrow 1922: 297

#### Materials

- **Type status:** Other material. **Occurrence:** recordedBy: Ming Bai and Panpan Li; individualCount: 32; sex: 14 males, 18 females; occurrenceID: 4F310F7A-F939-509A-B7FD-FC2CD45FFE53; **Location:** country: China; stateProvince: Guangdong; county: Wengyuan; locality: Chebaling National Nature Reserve; verbatimCoordinates: 24.7025°N, 114.2550°E; **Event:** year: 2022; month: 8

#### Distribution

**Figure 4.**
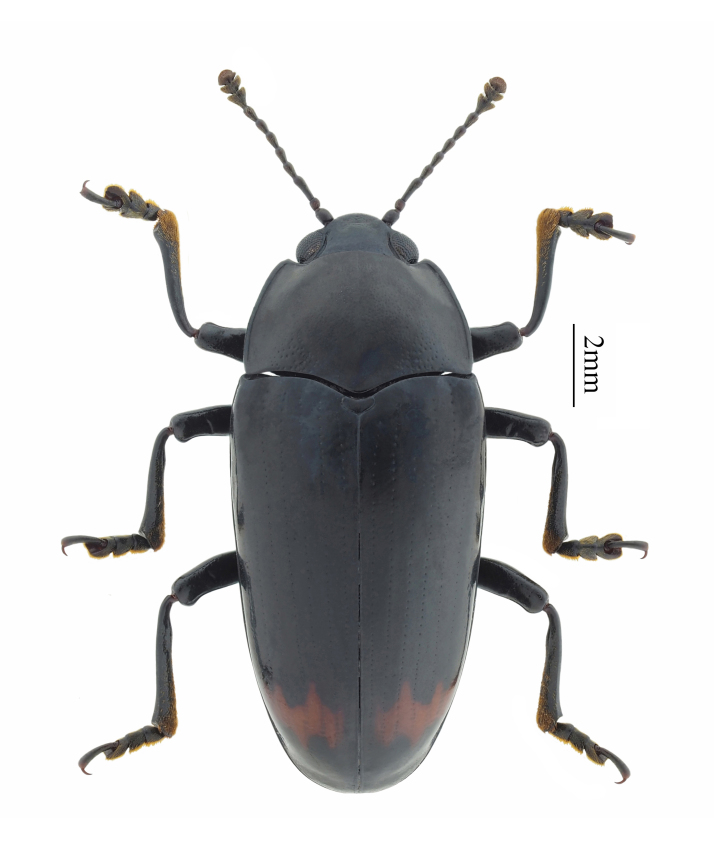
Micrencaustes (Micrencaustes) navicularis Arrow, 1922. Specimen from Guangdong, China.

Micrencaustes (Micrencaustes) navicularis Arrow, 1922 (Fig. 4) is recorded in Chebaling National Nature Reserve in Wengyuan County, Shaoguan City, Guangdong Province, China and is also distributed in Laos and Vietnam (Arrow 1922, Chûjô and Chûjô 1989).

## Identification Keys

### The key to species of the genus *Micrencaustes* from China

**Table:** 

1	Postmesocoxal lines absent	2
–	Postmesocoxal lines present	6
2	Pronotum and elytra with orange marks	3
–	Only elytra with orange marks	5
3	Pronotum with two marks	4
–	Pronotum with three marks	*M.occulta* **sp. nov.**
4	Each elytron with one mark	*M.liturata* (MacLeay)
–	Each elytron with two marks	*M.divisa* Arrow
5	2.4 times interocular distance as eye radius; maxillary terminal palpomere nearly 3.0 times as wide as long	*M.episcaphoides* Heller
–	1.5 times interocular distance as eye radius; maxillary terminal palpomere nearly 4.5 times as wide as long	*M.michioi* Osawa & Chûjô
6	Body without marks	*M.dehaanii* (Castelnau)
–	Body with obvious marks	7
7	Elytra without marks	8
–	Elytra with marks	9
8	Head without orange marks; prosternal lines exceeding the front edge of coxae	*M.acridentata* Li & Ren
–	Head with orange marks; prosternal lines reaching the front edge of coxae	*M.renshii* Meng, Ren & Li
9	Pronotum with marks	10
–	Pronotum without marks	11
10	The anterior edge of basal mark of elytra connected with the anterior edge of elytra	*M.lunulata* (Macleay)
–	The anterior edge of basal mark of elytra not connected with the anterior edge of elytra	*M.taiwana* Araki
11	Each elytron with a mark	*M.navicularis* Arrow
–	Each elytron with two marks	12
12	Abdomen with very large punctures along the outside edge of ventrite V	*M.decipiens* Arrow
–	Abdomen without very large punctures along the outside edge of ventrite V	13
13	Basal mark of elytron with two black dots near the anterior edge	*M.biomaculata* Meng, Ren & Li
–	Basal mark of elytron without black dots	*M.wunderlichi* Heller

## Supplementary Material

XML Treatment for Micrencaustes (Mimencaustes) occulta

XML Treatment for Micrencaustes (Mimencaustes) divisa

XML Treatment for Micrencaustes (Micrencaustes) navicularis
